# Epigenetics of Peripheral B-Cell Differentiation and the Antibody Response

**DOI:** 10.3389/fimmu.2015.00631

**Published:** 2015-12-14

**Authors:** Hong Zan, Paolo Casali

**Affiliations:** ^1^Department of Microbiology and Immunology, University of Texas School of Medicine, UT Health Science Center, San Antonio, TX, USA

**Keywords:** AID, B cell, Blimp-1, class switch DNA recombination, epigenetics, histone post-translational modification, memory B cell, microRNA, plasma cell differentiation, somatic hypermutation

## Abstract

Epigenetic modifications, such as histone post-translational modifications, DNA methylation, and alteration of gene expression by non-coding RNAs, including microRNAs (miRNAs) and long non-coding RNAs (lncRNAs), are heritable changes that are independent from the genomic DNA sequence. These regulate gene activities and, therefore, cellular functions. Epigenetic modifications act in concert with transcription factors and play critical roles in B cell development and differentiation, thereby modulating antibody responses to foreign- and self-antigens. Upon antigen encounter by mature B cells in the periphery, alterations of these lymphocytes epigenetic landscape are induced by the same stimuli that drive the antibody response. Such alterations instruct B cells to undergo immunoglobulin (Ig) class switch DNA recombination (CSR) and somatic hypermutation (SHM), as well as differentiation to memory B cells or long-lived plasma cells for the immune memory. Inducible histone modifications, together with DNA methylation and miRNAs modulate the transcriptome, particularly the expression of activation-induced cytidine deaminase, which is essential for CSR and SHM, and factors central to plasma cell differentiation, such as B lymphocyte-induced maturation protein-1. These inducible B cell-intrinsic epigenetic marks guide the maturation of antibody responses. Combinatorial histone modifications also function as histone codes to target CSR and, possibly, SHM machinery to the *Ig* loci by recruiting specific adaptors that can stabilize CSR/SHM factors. In addition, lncRNAs, such as recently reported lncRNA-CSR and an lncRNA generated through transcription of the S region that form G-quadruplex structures, are also important for CSR targeting. Epigenetic dysregulation in B cells, including the aberrant expression of non-coding RNAs and alterations of histone modifications and DNA methylation, can result in aberrant antibody responses to foreign antigens, such as those on microbial pathogens, and generation of pathogenic autoantibodies, IgE in allergic reactions, as well as B cell neoplasia. Epigenetic marks would be attractive targets for new therapeutics for autoimmune and allergic diseases, and B cell malignancies.

## Introduction

Epigenetic changes brought about by genetic susceptibility and/or environmental exposure can modulate gene expression and alter cellular functions without altering genomic sequences (1). Epigenetic modifications and factors, such as DNA methylation, histone post-translational modifications and non-coding RNAs, such as microRNAs (miRNAs) and long non-coding RNAs (lncRNAs), comprise the epigenome and interact with genetic programs to regulate immune responses. Immunoglobulin (Ig) class switch DNA recombination (CSR) and somatic hypermutation (SHM) are critical for the production of protective antibodies against microbial pathogens, IgE in allergic responses, as well as pathogenic autoantibodies in autoimmune diseases. CSR recombines S region DNA located upstream of constant heavy-chain (C_H_) region exons, thereby encoding new Ig C_H_ regions that endow antibodies new biological effector functions (2). SHM introduces mostly point mutations in variable regions of Ig heavy and light chains, thereby providing the structural substrate for antigen-mediated selection of B cell mutants with higher affinity B cell receptors (BCRs) (3–5). CSR and SHM occur mainly in germinal center and require activation-induced cytidine deaminase (AID, encoded by *AICDA* in humans and *Aicda* in mice), which is expressed in a differentiation stage-specific fashion in B cells (2–4). Class switched and hypermutated B cells further differentiate into long-lived memory B cells, which can react quickly to a recurrent antigenic challenge, or antibody-secreting plasma cells in a fashion critically dependent on B lymphocyte-induced maturation protein 1 (Blimp-1, encoded by *PRDM1* in humans and *Prdm1* in mice) (6, 7). Epigenetic modifications and factors influence gene expression and modulate critical B cell processes, such as CSR, SHM, and differentiation to memory B cells or plasma cells, thereby informing the antibody response (4, 8–10). Epigenetic dysregulation can result in aberrant antibody responses to exogenous antigens or self-antigens, such as chromatin, histones, and double-strand DNA in lupus.

B cell development and differentiation occur in two sequential stages. The initial, antigen-independent stage occurs in the bone marrow and involves recombination activating gene (RAG)1/RAG2-dependent V-(D)-J DNA rearrangement, which produces clonally unique Ig variable regions that specifically bind antigen. This stage generates mature, immunocompetent B cells that can bind to a unique antigen. The B cells move into the periphery and complete further, antigen-independent maturation into immunocompetent naïve mature B cells. In the periphery lymphoid organs, B cell undergoes the antigen-dependent stage of development or differentiation, upon activation by antigen binding and co-stimulation (5). In this stage, resting naïve mature B cells are induced to undergo cell proliferation, CSR, as well as SHM-mediated antibody affinity maturation, and differentiate into memory B cells, or short- or long-lived antibody-secreting plasma cells (6, 7). Multiple epigenetic changes are associated with each B cell development and differentiation stage. Resting, naïve B cells undergo V_H_DJ_H_-Cμ transcription, which initiates at the V_H_ promoter and runs through the intronic Sμ region and Cμ/Cδ exon clusters. This encodes the surface BCR, which comprises *Ig*μ** and *Ig*δ** heavy chain genes. These resting B cells display low levels of overall histone acetylation and genome-wide DNA hypermethylation, therefore most regions within the Ig heavy chain (*IgH*) locus are in a closed chromatin state (11), enriched in repressive histone post-translational modifications (e.g., H3K9me3 and H3K27me3) but lacking of activated histone modifications (12, 13). In B cells, epigenetic marks, such as DNA methylation, histone modifications, and miRNAs, are induced by the same stimuli that drive the antibody response, and modulate the transcriptome, especially the expression of AID, which is essential for SHM and CSR, and factors critical for plasma cell differentiation, such as Blimp-1 (4). By functioning as histone codes, combinatorial histone modifications also play a role in the targeting of the CSR and, possibly, SHM machinery to the *Ig* loci through recruiting specific scaffold proteins that stabilize CSR/SHM factors (8). These inducible B cell-intrinsic epigenetic marks control transcription programs that distinguish individual stages of B cell differentiation and underpin the molecular changes that are necessary for antibody response.

In this review, we provide a conceptual framework to understand how epigenetic modifications/factors modulate CSR and SHM, and the generation of plasma cells and memory B cells, with focus on AID-dependent peripheral B cell differentiation into memory B cells and long-lived plasma cells (but not differentiation of naïve B cells to short-lived plasma cells). We also highlight our current understanding of epigenetic modulations of CSR, SHM, and plasma cell differentiation by histone deacetylases (HDACs) inhibitors (HDIs). Finally, we summarize recent discoveries that indicate the importance of B cell epigenetic dysregulation in autoimmunity and B cell neoplasia.

## Epigenetic Regulation of AID Induction

Somatic hypermutation and CSR are initiated by transcription through V(D)J and the donor/acceptor S regions that will undergo recombination, respectively, and are mediated by AID, a 198 amino acid protein, which is structurally and functionally similar to apolipoprotein B RNA-editing cytidine deaminases (APOBEC enzymes) (2, 3). AID shares a conserved catalytic domain with other members of the APOBEC family of cytosine or cytidine deaminases (3). It deaminates deoxycytidines (dCs) into deoxyuracils (dUs) yielding dU:dG mismatches. These mismatches can be repaired by an error-prone DNA repair pathway, which introduce somatic mutations, or processed by uracil DNA glycosylase (Ung), which is recruited to and stabilized on S regions by the scaffold functions of 14-3-3 adaptors, the translesion DNA synthesis (TLS) polymerase Rev1 and replication protein A (RPA), or elements of the mismatch repair (MMR) machinery, such as Msh2 and Msh6, which trigger DNA repair processes leading to introduction of point mutations (SHM) or double-strand DNA breaks (DSBs) (CSR) (2).

**Figure 1:**
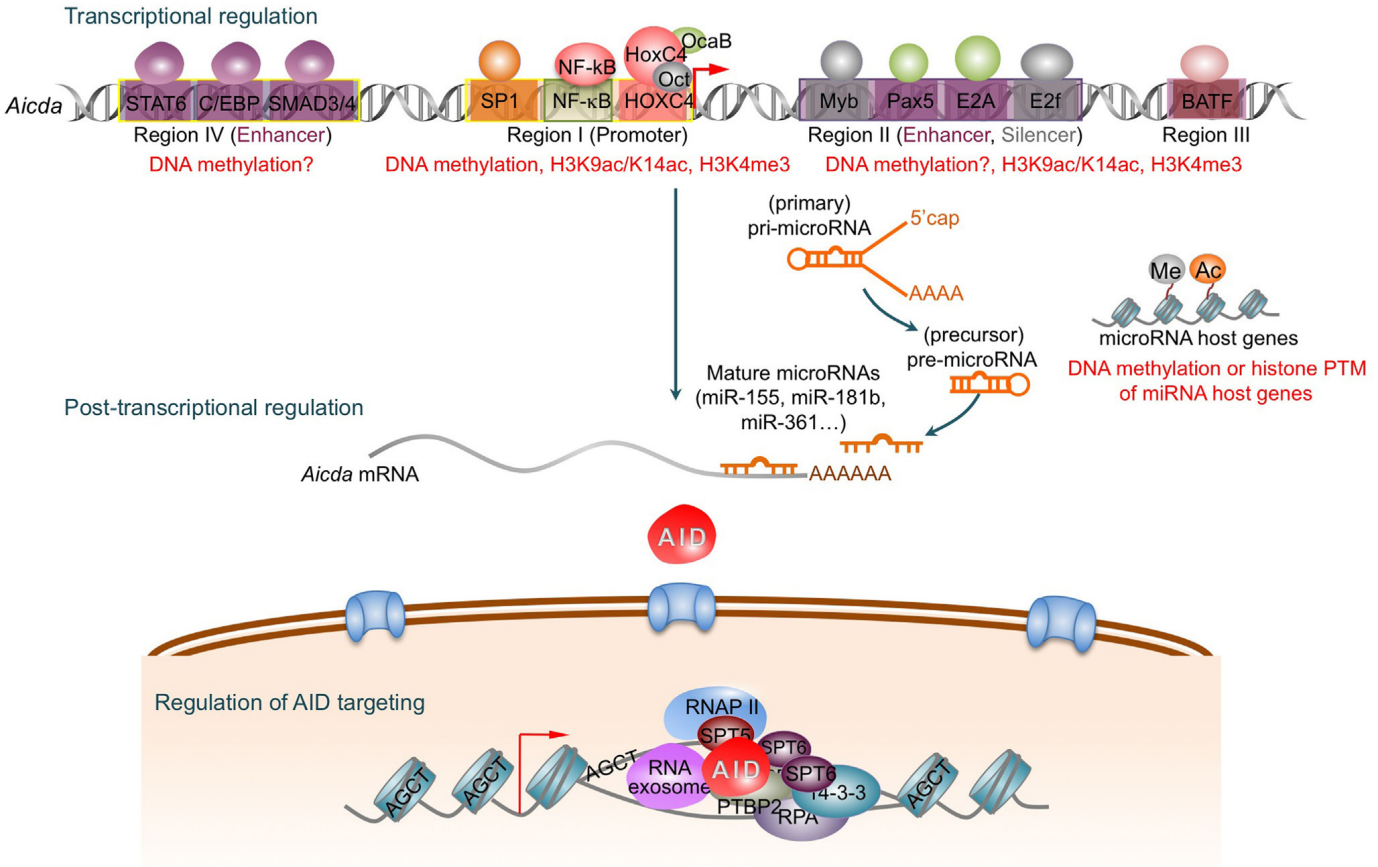
**AID is tightly regulated at the transcriptional, post-transcriptional, and post-translational levels**. Four distinct DNA regions (regions I–IV) of the AID gene (*Aicda*) locus possess binding sites for multiple transcription factors to regulate *Aicda* expression. Region I functions as promoters containing the binding sites for HoxC4/Oct and NF-κB/Sp1/Sp3, which can be induced by activating the *Aicda* promoter. In resting naive and memory B cells, and non-B cells, silencer elements in region II bind the repressor proteins E2f and c-Myb to counter the activity of the transcriptional activators. Stimulation of B cells with the primary inducing stimuli and cytokines that promote CSR induce activation signals through region IV enhancer in collaboration with the intronic enhancer in region II can overcome the effect of the region II silencer. After transcription, the *Aicda* mRNA can be negatively regulated by miR-155, miR-181b, and miR-361, which specifically bind to the conserved target sites on the 3′ UTR of *Aicda* mRNA. Nuclear AID is either degraded or exported back to the cytoplasm. Only a small proportion of AID molecules are targeted onto DNA at *Ig* or non-*Ig* loci by its co-factors. AID preferentially deaminates single-strand DNA, which emerges from transcription by RNA Pol II and depends on histone modifications in the transcribed locus. AID can be recruited to the open DNA before or during transcription. In CSR, AID recruitment to S regions occurs with interaction of RNA Pol II and AID-interacting factors, such as Spt5, Spt6, PTBP2, RNA exosome, lncRNAs, and 14-3-3 adaptor proteins, which would form a macromolecular complex. AID is enriched and stabilized on the targeted DNA by 14-3-3 adaptor proteins, which access the same S regions as the transcription machinery owing to their open chromatin state. These 14-3-3 adaptors are recruited and/or stabilized through interactions with 5′-AGCT-3′ repeats and possibly by H3K9acS10ph. The RNA exosome also interacts with AID and allows AID to deaminate both the transcribed and the non-transcribed DNA strand in the S regions undergoing transcription. AID deaminate dCs into dUs to yield dU:dG mismatches. Resolution of these lesions can lead to different physiological or pathological outcomes.

As a potent mutator, AID can effectively introduce mutations in not only *Ig* loci but also a variety of non-*Ig* genes, thereby causing genome instability in both B cells and non-B cells, including non-lymphoid cells, and contributing to tumorgenesis (3). Tight regulation of AID expression and function is necessary to maintain genomic stability in both B cells and non-B cells, and to avoid damages, such as chromosomal translocations, resulting from its dysregulation (14–18). This is achieved through fine control of transcription, post-transcription and post-translation regulation, nuclear/cytoplasmic distribution, stability, and activity (3) (Figure 1). AID express at a very low level (mostly undetectable) in naïve B cells, it is greatly induced in B cells undergoing SHM/CSR, and repressed in memory B cells and plasma cells to preserve the specificity, affinity, and isotype of antibody and BCR. *Aicda* transcription is under the control of multiple elements, particularly Homeobox protein C4 (HoxC4) (Figure 1). HoxC4 is a highly conserved helix-loop-helix homeodomain-containing transcription factor. As we have shown, HoxC4 directly binds to the *Aicda* promoter through an evolutionarily conserved 5′-ATTT-3′ site embedded within a conserved binding site for POU domain-containing transcription factors Oct1 and Oct2 (5′-ATTTGAAT-3′) (19). Sp1/Sp3 and NF-κB also bind the same promoter core and synergize with HoxC4 for *Aicda* induction (19, 20).

### Regulation of AID Expression by DNA Modifications and Histone Modifications

The expression of *Aicda* is modulated by changes of *Aicda* epigenetic status. Suppression of *Aicda* expression in naïve B cells is mediated by promoter DNA hypermethylation (21). In naive B cells, in which AID is not expressed, histone H3 acetylation occurs in the *Aicda* gene at low levels comparable to the overall H3 acetylation in the genome and neighboring genes, such as *Mfap5*, which is not significantly expressed in B cells (22). Upon activation of B cells, DNA of the *Aicda* gene is demethylated and the locus becomes enriched in H3K4me3 and H3K9ac/K14ac. B cell activation by LPS plus IL-4, which induces *Aicda* transcription, greatly increases H3 acetylation at the *Aicda* locus, particularly in the *Aicda* regulatory regions (22). These epigenetic modifications, together with induction of nuclear factor NF-κB, homeobox protein HoxC4 and other transcription factors, activate *Aicda* transcription. Transcription elongation depends on the H3K36me3 posttranslational modicification, an intragenic mark of gene activation in the *Aicda* gene body, as suggested by AID down-regulation following depletion of the H3K36me3 methyltransferase Setd2 (3, 23). Post-CSR/SHM down-regulation of *Aicda* transcription probably results from remethylation of the *Aicda* DNA.

### Regulation of AID Expression by miRNA

In addition to DNA methylation of the *Aicda* promoter and histone acetylation of the *Aicda* locus, selected miRNAs provide a more important mechanism of modulation of AID expression. miRNAs are small (~21–23 nt), evolutionarily conserved non-coding RNAs derived from much larger primary transcripts encoded by their “host genes.” miRNAs bind to complementary sequences within the 3′ untranslated region (3′ UTR) of their target mRNAs and negatively regulate protein expression at the post-transcriptional level. miRNA can recognize its target mRNA through the short (as few as 6–8 nt) “seed sequence” at the 5′ end of the miRNA. miRNA repress gene expression by accelerating mRNA degradation and inhibiting mRNA translation. A given miRNA can potentially target different mRNAs, and a given mRNA can be targeted by multiple miRNAs. The mammalian genome encodes hundreds of miRNAs that collectively impact the expression of about one-third of all genes. miRNAs are transcribed from intergenic genomic DNA by RNA polymerase (Pol) II or Pol III as primary transcripts (pri-miRNAs) that are then cleaved by the nuclear ribonuclease Drosha to generate an about 70 bp long characteristic stem-loop structure, known as a pre-miRNA, and exported to the cytoplasm (24). The pre-miRNA is further processed by cytoplasmic enzyme Dicer into mature miRNA (24), which forms complexes with ribonucleoproteins (RNPs) and expresses regulatory effects (25, 26).

microRNAs control various biological processes by fine-tuning gene expression at the post-transcriptional level. They can modulate a wide range of physiological and pathological processes by regulating cellular function at every aspect, from proliferation and apoptosis to differentiation. miRNAs are present in all human and mouse cells, in which they each modulate the expression of a few to hundreds of target genes. miRNAs likely play important roles in B cell development and peripheral differentiation, as well as T cell stage-specific differentiation and autoimmunity. Naïve B cells, germinal center B cells, memory B cells, and plasma cells show distinct miRNA expression profiles (27, 28). Deletion of Dicer, which is critical for miRNA maturation, in activated B cells resulted in defective generation of germinal center B cells, memory B cells, and plasma cells (29). Indeed, Dicer-deficient B cells showed impaired biogenesis of many miRNAs, including miR-155 and miR-125b, which regulate expression of genes that modulate B cell germinal center reaction and plasma cell differentiation (30, 31). A single miRNA, such as miR-155, can target multiple genes, including *Myd88*, *Pu.1*, and *Aicda*, and regulate sequential stages of B cell differentiation (30, 32–34). By contrast, multiple miRNAs, such as miR-15a and miR-16, can cooperatively repress one critical gene in germinal center B cells, *Bcl2* (27).

miR-155, miR-181b, and miR-361 modulate AID expression by binding to the evolutionarily conserved target sites in the 3′ UTR of *AICDA/Aicda* mRNA, thereby reducing both *AICDA/Aicda* mRNA and AID protein levels (33–37). These miRNAs could suppress AID expression in naïve B cells and in B cells that completed SHM/CSR. miR-155 is the most abundant miRNA that has been shown to silence AID expression. miR-155 is processed from an RNA sequence encoded by miR-155 host gene (*miR155HG*). This was originally identified as a gene transcriptionally activated by promoter insertion at a common retroviral integration site in B-cell lymphomas (*Bic*, B-cell integration cluster). *Bic* RNA is a spliced and polyadenylated but non-protein-coding RNA that accumulates in lymphoma cells (32) and is induced along with AID in B cells activated by CSR-inducing stimuli (33, 34). The sequences of pre-miRNA-155 and mature miR-155 are highly conserved across more than 22 different organisms, including mammals, amphibians, birds, reptiles, sea squirts, and sea lampreys (38).

miR-155 and miR-361 are directly repressed by BCL-6, a transcriptional repressor required for germinal center formation. miR-361 is embedded in the *CHM* gene, which encodes a subunit of a Rab geranylgeranyl transferase and is known for its genetic inactivation in choroideremia (37). BCL-6 display a coordinated activity in sustaining high levels of AID expression in germinal center B cells undergoing CSR and SHM. By direct repressing miR-155 and miR-361, BCL-6 upregulates the expression of the target genes of these miRNAs, including *AICDA* and other elements involved in the maintenance of the germinal center B cell centroblast phenotype (37). The specific effect of miR-155 in the regulation of AID expression was demonstrated by the findings that disruption of the miR-155 binding site in the 3′ UTR of *Aicda* mRNA in B cells led to an increase in *Aicda* mRNA and AID protein by increasing the half-life of *Aicda* mRNA, resulting in increased CSR and *c*-*Myc/IgH* translocations (33, 34). Nevertheless, the role of miR-155 in regulating B cell function and antibody response is much more than modulating the expression of AID. miR-155 is expressed at a high level in germinal center B cells and plays an important role in germinal center formation and subsequent antibody response following antigen challenge (30, 32, 39). miR-155 deficiency in B cells resulted in reduced extra follicular and germinal center responses, decreased numbers of IgG1^+^ plasma cells and memory B cells, and failed production of high-affinity IgG1 antibodies (30). In B6/*Fas^*lpr/lpr*^* mice, deficiency of miR-155 results in a reduced autoantibody production and autoimmunity (40). This is likely stemmed from dysregulation of a variety of genes in multiple immune cells, including derepressed expression of SHIP-1 in B cells, which lead to mitigation of B-cell activation, proliferation and autoantibody production (40).

The 3′ UTR of *Aicda* mRNA contains multiple putative binding sites for miR-181b, which is predominantly expressed in lymphoid cells (10, 41). miR-181b is expressed at the highest levels in resting B cells and is downregulated upon B cell activation by CSR-inducing stimuli (34, 42). Expression of miR-181b in B cells leads to down-regulation of AID at both the transcript and protein levels. It has been suggested that miR-181b and miR-155 have non-overlapping functions in controlling AID expression. miR-181b may inhibit premature AID activity but allows proper AID transcriptional activation at early time points, while miR-155 could narrow AID function at a later stage of activation (34). By controlling AID expression, miR-155 and miR-181b protect resting B cells and non-B cells from AID-mediated mutagenesis. Accordingly, in Burkitt’s lymphoma B cells, deficiency of miR-155 expression is associated with high levels of somatic mutations and inter-chromosomal translocations (43).

## Epigenetic Regulation of AID Targeting in CSR and SHM

One fundamental question for B cell biology remains to be answered is how CSR and SHM machineries are targeted to the *Ig* locus. For CSR to take place, the *IgH* genes are subjected to transcriptional activation, RNA splicing, AID-mediated cytidine deamination, as well as DNA cleavage, repair, and recombination. Each of these events is likely associated with, and possibly regulated by specific changes in chromatin structure. Histone modifications in S and V(D)J regions are critical for targeting of the CSR and SHM machinery, respectively (Table 1). Chromatin structure that impacts on and likely regulate most aspects of gene expression, also contributes to the regulation of CSR and SHM. In B cells poised to undergo CSR, the *IgH* genes are in an “accessible” chromatin conformation before CSR (44). Upon induction of germ-line transcription, histones H3 and H4 have been shown to be acetylated at the I_H_ exon promoters and S regions (45, 46).

**Table 1:** **Epigenetic marks/factors, and their functions in CSR and SHM**.

Target(s)	Epigenetic mark(s)	(Putative) functions	Modulate		Reference
			CSR	SHM	
**microRNAs**					
*Aicda*	miR-93	Decrease expression of AID	+	+	(9, 33–37)
	miR-155				
	miR-181b				
	miR-361				
**lncRNAs**					
S regions	Germline I_H_-S-C_H_ transcripts	Increase S region accessibility	+	−	(2)
S regions	Intronic switch RNA	Recruit AID to S region	+	−	(69)
S regions	Antisense S region transcripts	Increase S region accessibility	+	−	(173)
V_H_DJ_H_	Antisense V_H_DJ_H_ transcripts	Increase V_H_DJ_H_ region accessibility	−	+	(173)
IgH 3′ RR super-enhancer	lncRNA-CSR	Regulate IgH 3′ regulatory region super-enhancer function	+	−	(70)
IgH and other AID target regions	xTSS-RNAs	Recruit AID to ssDNA-forming site	+	+	(67)
**DNA METHYLATION**					
V(D)J	DNA hypomethylation	Increases V(D)J region accessibility	−	+	(84)
*Igh* 3′-LCR	DNA hypomethylation	Mediates germline V_H_DJ_H_ and I_H_-S-C_H_ transcription	+	+	(174)
**HISTONE MODIFICATIONS**					
V(D)J	H3K4me2/3, H3K9ac/K14ac, H4K8ac	Increase V(D)J region accessibility and transcription	−	+	(23, 72, 85)
iEμ	H3K4me3, H3K9ac/K14ac	Activate iEμ and enhance germline VDJ transcription and I_H_-S-C_H_ transcription	+	+	(46, 47)
*Igh* 3′-LCR	H3K4me1/2	Mediate VDJ and germline transcription	+	+	(175)
	H3K9ac, H3K27ac, H4K8ac, H2BK5ac				(47)
S region(s)	H3K27me3	Decreases S region(s) accessibility	+	−	(12)
	H3K4me3, H3K9ac/K14ac, H3K27ac, H4K8ac	Increase S region(s) accessibility	+	−	(12, 13, 45–49, 61, 176)
	H3K9me3	Recruits the HP1γ-KAP1 complex and AID to Sμ region	+	−	(13)
	H3K9acS10ph	Recruits 14-3-3 adaptors and AID to S region(s)	+	−	(8)
	H4K20me2	Recruits 53BP1 to S region(s) in the DNA repair stage	+	−	(62)
*Aicda*	H3K4me3, H3K9ac/K14ac, H3K36me3	Increase transcription of *Aicda*	+	+	(22, 47)

*Repressive histone methylation: H3K9me3, H3K27me3*.

*Activating and recruiting histone modifications include histone acetylation: H3K9ac, H3K14ac, H3K27ac, H4K8ac, H2BK5ac, and H3K9ac; histone methylations: H3K4me2/3, H4K20me2, and H3K36me3; combinatorial histone H3K9acS10ph modification; histone phosphorylation: H2BS14ph; histone ubiquitination: H2AK119ub and H2BK120ub*.

### Epigenetic Targeting of the CSR Machinery

#### Histone Modifications in CSR Targeting

Histone post-translational modifications are important for targeting of the CSR machinery to the upstream donor and the downstream acceptor S regions that are involved in CSR (Table 1). The significant levels of germline Iμ-Sμ-Cμ transcription and activating histone marks, such as H3K4me3, H3K36me3, H2BK5ac, H3K9ac/K14ac, H3K27ac, and H4K8ac, are observed in the donor Sμ region even in resting naïve B cells, suggesting that Sμ is in a constitutively open state and poised for switch recombination (8). CSR induction requires both primary and secondary (CSR-inducing) stimuli. Primary stimuli, such as engagement of B cell CD40 by CD154 expressed on activated T cells, and T-independent dual toll-like receptor (TLR) and BCR engagement, induce B cell activation, proliferation, and differentiation. In conjunction with primary stimuli, secondary stimuli, which comprise cytokines (e.g., IL-4, TGF-β, and IFN-γ), direct class switching to IgG, IgA, or IgE by selecting the acceptor S region, through activation of S region histone modifications and inducing specific germline I_H_-S-C_H_ transcription (46–49). Primary stimuli induce histone-modifying enzymes and trigger chromatin decondensation in downstream S regions by removing repressive H3K9me3 and H3K27me3. These allow histone-modifying enzymes to ride on the RNA Pol II to reach S regions during germline I_H_-S-C_H_ transcription elongation (46, 47, 50–52). Upon RNA Pol II stalling, caused by complex secondary DNA structures, such as cruciform-like structures and R-loop, in S regions (2, 53), histone-modifying enzymes catalyze histone modifications in these regions. DNA transcription together with modified histones opens the chromatin in S regions, thereby allowing for access of CSR factors. The role of histone-modifying enzymes in CSR is further emphasized by the reduced S region histone modifications and CSR in B cells knockdown H3K4 methyltransferase Set1 (48), or H3K9 acetyltransferases Pcaf and Gcn5 (8).

In the donor and acceptor S regions, activating histone modifications, such as H3K9ac and H3K4me3, are enriched at levels much higher than those in the associated I_H_ promoter and C_H_ regions (46, 47). These histone marks are read by CSR factors, including 14-3-3 adaptors, which, such as AID and histone-modifying enzymes, are induced by primary CSR stimuli (54). 14-3-3 adaptors directly interact with AID and target it to the upstream and downstream S regions that undergo recombination (2), thereby transducing the epigenetic code. 14-3-3 adaptors specifically bind to the combinatorial histone H3K9acS10ph modification in S regions and 5′-AGCT-3′ (2, 54) repeats. These are characteristic motifs of all *IgH* locus S regions, but not I_H_ promoters, C_H_ regions or other genome areas (54). Due to their high affinity for 5′-AGCT-3′ repeats, 14-3-3 can potentially bind all S regions. Nevertheless, these adaptors are recruited only to the S regions that undergo recombination (54). This is due to the open chromatin state of such regions as well as specific binding of 14-3-3 to H3K9acS10ph (8). The specificity of 14-3-3 adaptors for H3K9acS10ph and 5′-AGCT-3′ repeats are evocative of RAG1/RAG2 complex specificity for H3K4me3 and V, D and J gene recombination signal sequences (RSSs) (55).

H3K4 methylation, particularly H3K4me3, plays a critical role in AID-mediated DNA cleavage in S regions during CSR (48). The formation of H3K4me3 at AID target loci is dependent on the histone chaperone complex, facilitates chromatin transcription (FACT), a chromatin-modifying complex during RNA processing (48, 56). We have shown that specific H3K4 methyltransferases and H3K9 acetyltransferases can be induced by TLR or CD40 signaling and catalyze histone H3K4me3 and H3K9ac/K14ac modifications. These are decorated S regions, regardless of whether they are targets of CSR (8). Conversely, the combinatorial histone H3K9acS10ph modification specifically marks the S regions set to recombine and directly recruits 14-3-3 adaptors for AID stabilization (8). 14-3-3 adaptors, which possess no enzymatic activity, function as histone code readers to recruit/stabilize downstream effector molecules, which *per se* cannot read histone codes, consistent with the “histone code hypothesis” (57, 58). Inhibition of the enzymatic activity of Gcn5/Pcaf histone acetyltransferases leads to decreased H3K9acS10ph, 14-3-3 recruitment and AID stabilization in S regions, and CSR.

H3K9me3 is also present, although at a relatively low level, in Sμ but not downstream S regions. In the Sμ region, H3K9me3 recruits the KAP1-HP1γ complex to stabilize AID (13). Accordingly, CSR to IgA can be impaired by deletion of the H3K9 methyltransferase Suv39 (59). Histone modification readers that function as scaffolds, such as 14-3-3 adaptors, translesion DNA synthesis polymerase Rev1 (60) and RPA (61), act as core for the assembly of macromolecular complexes on S region DNA, to stabilize AID and/or Ung for generation of DNA lesions. Histone modifications are also recognized by DNA repair factors, such as p53-binding protein 1 (53BP1), which may functions as a scaffold to recruit/stabilize additional DNA repair factors for CSR. Abrogation of histone methyltransferase MMSET expression impairs H4K20me2 enrichment and 53BP1 recruitment in S regions, and results in reduced CSR (62).

Suppressor of Ty6 (Spt6), a RNA Pol II-interacting histone H3-H4 chaperone, also plays a role in the regulation of H3K4me3 for SHM and CSR (23). Depletion of Spt6 impaired H3K4me3 and AID-mediated DSBs in the S regions in CH12 B cells, which can otherwise be induced to express AID and CSR to IgA (23). In addition, knockdown Spt6 in human Burkitt’s lymphoma BL2 cells overexpressing mutant AID (JP8Bdel) that lacks C-terminal 16 aa residues, which are critical for CSR but not to SHM (63), abolished SHM and H3K4me3 in *Ig* V_H_ region and non-*Ig* AID target genes (23). Thus, activating histone modifications are induced in S regions and create an open chromatin state, which allows for the access of the CSR machinery. Epigenetic specification of CSR targeting entails reading of histone codes by scaffold proteins, which orchestrate the assembly of macromolecular complexes in the sequential DNA lesion and repair stages.

#### Long Non-Coding RNA and CSR Targeting

Long non-coding RNAs are evolutionarily conserved non-coding RNA molecules that are longer than 200 nt and located within the intergenic loci or regions overlapping antisense transcripts of protein coding genes (64–66). Their expression can be tissue- and cell-type specific. lncRNAs are involved in numerous cellular functions, such as transcriptional regulation, RNA processing, RNA modification and epigenetic silencing. lncRNAs have been recently shown to play an important role in the targeting of the CSR machinery (Table 1). They target AID to divergently transcribed loci in B cells (67). In B cells undergoing CSR, the RNA exosome, a cellular RNA-processing/degradation complex is required for optimal CSR (68). The RNA exosome associates with AID, accumulates on S regions in an AID-dependent fashion. Both the cellular RNA exosome complex and a recombinant RNA exosome core complex inform robust transcription-dependent DNA deamination by AID in both strands of transcribed SHM substrates *in vitro*. In B cells, deficiency of *Exosc3* or *Exosc10*, the essential subunits of the RNA exosome complex, impairs CSR and SHM (67, 69). Many novel RNA exosome substrate lncRNAs have been identified by transcriptome analysis of *Exosc3*- or *Exosc10-*deficient B cells. RNA exosome-regulated, antisense-transcribed regions accumulate single-strand DNA structures containing RNA-DNA hybrids and recruit AID in B cell. RNA exosome regulation of lncRNA has been suggested to recruit AID to single-strand DNA-forming sites of antisense and divergent transcription in the B cell genome (67). In addition, a recent study has shown that an lncRNA generated by S region transcription followed by lariat debranching can fold into G-quadruplex structures, which can be directly bound by AID, thereby targeting of AID to S region DNA (70).

### Epigenetic Targeting of the SHM Machinery

Activation-induced cytidine deaminase initiates SHM by deaminating cytosine residues in Ig V(D)J genes. It also introduces DNA damages, including point-mutations, in non-*Ig* loci at a lower frequency. How AID is recruited to the target sites is not fully understood. AID has been suggested to target a specific microenvironment rather than a defined set of genes (71). The SHM machinery is targeted to the V(D)J region through unique targeting sequences, transcription, and possibly, DNA demethylation and histone modifications (Table 1) (72). Like CSR, SHM requires not only the expression of AID, but also transcription in the target regions. Alterations in chromatin structure at *IgH* may also play an important role in promoting and/or stabilizing AID targeting (73). AID targets a distinct set of hotspots, which are concentrated in genes that are highly transcribed but frequently stalled genes (74). AID associates with RNA Pol II (75). RNA Pol II transcription, as well as several chromatin alterations could facilitate the access of AID to *Ig* loci and give use to single-stranded DNA, a preferential substrate for AID deamination. However, transcription alone is insufficient to recruit AID activity (74). AID targets are predominantly grouped within topological complex, highly transcribed superenhancers and regulatory clusters, which are enriched in chromatin modifications associated with active enhancers (such as H3K27Ac), they are also and marks of active transcription (such as H3K36me3), indicating that these features are universal mediators of AID recruitment (71, 74, 76). In both human and mouse B cells, there is a strong overlap between hypermutated genes and superenhancer domains (71).

The phosphorylated histone H2B (H2BSer14P) correlates tightly with SHM and CSR. In Ig V(D)J and S regions, H2B phosphorylation requires AID and may be mediated by the histone kinase Mst1 (72, 77). It has been suggested that SHM and CSR trigger distinct DNA damage responses and identify a novel histone modification pattern for SHM consisting of H2B (Ser14P) in the absence of γH2AX (72, 77). The non-Ig AID targets share important characteristics with Ig genes, namely, repetitive sequences that can form non-B DNA structures upon efficient transcription, and the accumulation of chromatin H3K4me3 histone marks (78).

The FACT complex may also promote SHM (56). FACT, a histone chaperone-type elongation factor, was originally discovered for its biochemical activity to promote transcription elongation of RNAPII on the nucleosomal DNA template (79). It has been suggested to remove nucleosomal histones and deposit them at the RNAPII of transcription site, and this allows the RNA polymerase to be proceed beyond the nucleosomes (80). FACT is important for inducing H3K4me3, which can be recognized by a protein complex with DNA cleaving activity and accumulates at SHM-targeted genomic regions (48). Furthermore, FACT and histone variant H3.3, a hallmark of replication-independent histone turnover, are enriched at the heavy and light chain V(D)J regions, the light chain Jκ5 region and the Sμ region 5′ flanking sequence (48). The importance of the chromatin histone-exchanging dynamics in SHM target regions, especially Ig genes, is emphasized by high abundant FACT and H3.3 deposition in the most efficient targets of SHM (56, 81). Histone post-translational modifications would also mediate recruitment of DNA repair factors, such as error-prone TLS DNA polymerases, in SHM at the DNA repair stage. H2AK119 ubiquitination (ub) and H2BK120ub are enriched in V_H_DJ_H_ but not C_H_ regions and colocalize with DNA Pol η, an important TLS polymerase for SHM, in AID nuclear foci (82). Pol η is likely recruited to those loci by H2AK119ub and H2BK120ub, as well as by monoubiquitinated PCNA scaffold, through its ubiquitin-binding domain (83). As mentioned before, AID-dependent histone H2BSer14P mark in the V(D)J region (72) may also contributes to recruitment of DNA repair factors. The role of DNA hypomethylation in SHM has been suggested by the finding that only the hypomethylated allele is hypermutated in B cells carrying two nearly identical pre-rearranged Igκ transgenic alleles, even though transcription of both alleles are comparable (84).

It is possible that DNA demethylation facilitates SHM targeting by promoting histone modifications H3K4me3, H3K9ac/K14ac and H4K8ac, which are enriched in the V(D)J region and associated with an open chromatin state (23, 72, 85). Both H3K4me3 and H4K8ac are involved in SHM. Decreased H3K4me3 in V_H_DJ_H_ regions in human BL2 cells, a Burkitt’s lymphoma cell line that can be induced to undergo SHM, upon knockdown of histone chaperone Spt6 is associated with reduced V_H_DJ_H_ mutations (23). In BL2 cells, H4K8ac increases concomitantly with V_H_DJ_H_ mutations upon treatment with HDI trichostatin A (TSA) (85). Persistent H4K8ac in VλJλ region required E2A, whose inactivation result in decreased mutations (72, 86).

Some *cis*-acting regulatory regions, such as *Ig* enhancer and *Ig* enhancer-like sequences, are important for targeting SHM to *Ig* loci (87, 88). In chicken, mouse and human B cells that *Ig* locus enhancers and enhancer-like elements function as core diversification activator (DIVAC) sequences that work together to target SHM (88). In chicken DT40 B cells, short mammalian *Ig*λ and *IgH* enhancer fragments can increase mutation rates by more than 20-fold (88). lncRNAs, which are likely to regulate many biological functions, have been recently shown to be link to enhancer activity. lncRNAs are expressed in a lineage-specific fashion and function through RNA–protein, RNA–DNA, or RNA–RNA target interactions. They are induced to modulate innate and adaptive immunity (66). Many regulatory lncRNAs can be categorized as DNA accessibility modulators, and likely play a role in SHM targeting, especially in conjunction with the function of *Ig* enhancers (67). Thus, activating histone modifications, DNA hypomethylation, and possibly lncRNAs increase V(D)J region chromatin accessibility to the SHM machinery, including AID and error-prone DNA repair factors, which can be stabilized by modified histones.

## Epigenetic Regulation of Plasma Cell Differentiation

B cell differentiation is initiated by extracellular stimuli that bind to cellular receptors and trigger a signaling cascade resulting in the induction of transcription factors that reprogram B cells to secrete antibodies. The function of transcription factors is controlled by the accessibility to DNA through epigenetic modifications. Little is known about how the epigenetic mechanisms direct B cell differentiation into antibody-secreting plasma cells (6). Plasma cells are terminally differentiated elements in the B cell lineage that mostly have undergone SHM and CSR. These cells do not proliferate, but secrete large volumes (10^7^ molecules/h) of clone-specific antibodies. Plasma cells are derived from either germinal center or memory B cells. Although some memory B cells are IgM^+^, most memory B cells are class-switched and express mutated V(D)J gene segments. Upon reactivation by specific antigen, memory B cells differentiate into antibody-secreting plasma cells to mediate an anamnestic humoral response (89). Reactivated memory B cells can also reenter into the germinal center reaction and undergo further CSR and/or SHM before differentiating into plasma cells or reverting back to memory B cells (90, 91).

Plasma cells display a transcriptional signature that is distinct from B cells (92). Their changes in gene expression correlated with the acquisition of permissive histone modifications, including H3K4me1 and H3K4me4, which are enriched in active promoters and distal enhancers and play an important role in B cell development (92, 93). Upregulation of Blimp-1, a transcriptional repressor, is central to plasma differentiation (6). Blimp-1 down-regulates the expression of *Bcl6*, *Pax5*, and *Spib*, all of which inhibit B cell differentiation into plasma cells by binding to the promoters of these genes (6), and, possibly, deacetylating their promoters. Indeed, *Pax5, Spib*, and perhaps *Bcl6* promoters display decreased histone acetylation in plasma cells (94, 95). Furthermore, Blimp-1 can interact with HDACs that remove acetyl groups on a histone (94). In addition, in plasma cells, Blimp-1 down-regulates *c-Myc* expression through a similar epigenetic mechanism (94), thereby maintaining the terminal differentiation state of these cells (6). Finally, Blimp-1 can interact with H3K9 methyltransferase G9a and likely recruits this enzyme to the *Pax5* and *Spib* promoters, thereby increasing H3K9me3 and repressing activation of these promoters (95, 96). Thus, epigenetic induction of Blimp-1 and Blimp-1-mediated epigenetic inhibition of target genes drives plasma cell differentiation, and possibly maintains plasma cell identity. Down-regulation of Pax-5 and Pax-5-driven Bcl-6 lead to derepression of the *Prdm1* promoter from Bcl-6-mediated epigenetic silencing. This is associated with increased histone acetylation in the *Prdm1* promoter, likely resulting from release of Bcl-6-bound HDACs (97, 98). Reduction of Blimp-1 in a plasmacytoid cell line by enforced expression of Bcl-6 resulted in re-expression of B cell markers, including CD19 (98).

*Prdm1* mRNA contains a long (>2,000 nt) 3′ UTR, which can be potentially targeted by multiple miRNAs, including miR-9, miR-23b, miR-30, miR-125b, miR-127, and let-7 (10, 27, 28, 31, 99–102). Overexpression of miR-125b in B cells impairs expression of Blimp-1 and inhibits B cell differentiation into plasma cells (31). In addition, miR-125b can downregulate IFN regulatory factor (Irf)-4, which reciprocally regulates Blimp-1 and is required for the generation of plasma cells (27, 31, 103). Furthermore, X-box binding protein (Xbp)-1 that governs late events of plasma cell differentiation can be downregulated by miR-127 (102).

## Epigenetic Regulation of Memory B Cell Differentiation

B cell memory is a hallmark of adaptive immunity. Memory B cells are antigen-experienced quiescent B cells, which can be generated in response to both T-dependent antigens (usually proteins) and T-independent antigens (usually carbohydrates) (7). B cells quickly react to a second challenge with the same antigen, thereby providing humoral immune protection. While inherit epigenetic information from their active B cell precursors, memory B cells acquire new epigenetic marks, which make these resting B cells poised to quickly respond to experienced antigen. Expression of memory B cell hallmark genes, such as *CD27* (in humans) and *Cd38* (in mice), is likely mediated by histone modifications induced during B cell activation (4). Genes that control B cell identity and function, including *Pax5* and *Spib*, are also expressed in memory B cells, likely reflecting the epigenetic state that originated in naïve B cells and led them to memory B cells differentiation. Post-recombined S–S regions in the *IgH* locus show constitutive (upstream Sμ portion) or induced (downstream Sγ, Sϵ, or Sα portion) histone modifications, which could be transferred to memory B cells and result in comparable epigenetic landscapes in class-switched memory B cells (4). The functional distinction between memory B cells and their naïve counterparts could at least partially result from the epigenetic alterations.

Quiescent and activated B cells display different histone marks (104). In resting cells, histone lysine methylation was reduced as compared to activated cells (105). Enhancer of zeste homolog 2 (Ezh2) catalyzes H3K27me3, which is enriched at transcription start sites of repressed genes, through its SET domain. EZH2 is highly expressed in human germinal center B cells. Inactivation of Ezh2 in mouse germinal center B cells resulted in a profound reduction of germinal center reactions, memory B cell formation, and antibody response (106). Ezh2 protected germinal center B cells against AID mutagenesis and facilitated cell cycle progression. Repression of Blimp-1 and Irf4 expression in germinal center B cells is also necessary to limit plasma cell differentiation (106). In *Ezh2^*fl/fl*^*Cγ*1-Cre* mice, the B cell differentiation stage-specific Ezh2 deficiency resulted in profound impairment of germinal center reactions and memory B cell formation, suggesting that the methyltransferase activity of *Ezh2* is essential for not only germinal center B cell functions but also generation of memory B cells (106). Furthermore, it has been recently shown that histone acetyltransferase monocytic leukemia zinc finger protein (MOZ), which specifically targets H3K9 and plays a role in stem cell self-renewal, regulates B cell memory formation, controlling memory compartment composition (104). This activity of MOZ is B cell-intrinsic and is required for establishing the germinal center gene expression program. B cell stage-specific deletion of MOZ alters fate decisions in both primary and secondary antibody responses. The lack of MOZ affected the functional outcome of antibody responses, with an increase in secondary germinal centers and a corresponding decrease in secondary high-affinity antibody-secreting cell formation (104).

The differentiation of naïve B cells to germinal center B cells and then to plasma cells or memory B cells would also be associated with changes in DNA methylation. Several genes can be silenced by DNA methylation catalyzed by DNA methyltransferases (DNMTs), such as DNMT3a, which is highly expressed in memory B cells (107). In memory B cells, different expression, as compare to naïve B cells, of the immune activation related elements is likely concomitant with distinctive DNA methylation (108). This supports the concept that the memory B cell epigenome is poised to facilitate a more rapid and robust activation response than that of its naive counterparts.

As demonstrated by concomitant miRNA and mRNA profiling, miRNAs play a regulatory role at every stage of the B cell peripheral differentiation process (28). Selected miRNAs, including miR-125b and let-7, negatively regulate *Prdm1* (10, 27, 28, 101). Down-regulation of miR-15a and miR-16, which target *Bcl2*, likely contributes to memory B cell survival (27, 109) and re-expression of Krüppel-like factors (KLFs), which can bind HDACs, mediates memory B cell quiescence (110). miR-223 is enriched in human memory B cells and down-regulates the expression of LMO2, a key transcription factor in B cell differentiation (28). In addition to function as a negative regulator for CSR and SHM by silencing AID, miR-155 also plays an important role in memory B cell responses. miR-155 deficiency greatly reduced memory B cells (111). Several lncRNAs have been shown to be preferentially expressed in human memory B cells, but not in naïve B cells or B1 cells (112), suggesting that lncRNA also play a role in memory B cell differentiation. Thus, DNA methylation, histone modifications and non-coding RNAs, especially miRNAs, control gene expression programs that lead to B cell differentiation into plasma cells and memory B cells, and maintain the identity of these differentiated cells.

## Epigenetic Modulation of Aid and Blimp-1 Expression, CSR, SHM, and Plasma Cell Differentiation by HDAC Inhibitors

Histone deacetylases are a class of enzymes that remove the acetyl groups from the lysine residues on a histone leading to the formation of a condensed and transcriptionally silenced chromatin. HDIs block this action and can result in histone hyperacetylation, thereby affecting gene expression. HDIs have been shown to alter gene expression by altering chromatin accessibility (113–115). In immune cells, these epigenetic modifiers exert modulatory effects even at moderate concentrations. By using well-characterized short-chain fatty acid (SCFA) HDIs, valproic acid (VPA) (116), and butyrate (117), we have shown that HDIs regulate intrinsic B cell functions that are critical in shaping effective antibody and autoantibody responses. Our findings were further supported by a recent publication showing that HDIs Panobinostat (a novel broad-spectrum HDI) and Vorinostat (suberanilohydroxamic acid or SAHA) significantly impair antibody and autoantibody responses (118). VPA is an FDA-approved drug, widely used as an anticonvulsant and a mood-stabilizer. It selectively inhibits class I HDACs, particularly, HDAC1 and HDAC2, and, less effectively, class IIa HDACs, of the four HDAC classes identified in mammals (116, 119). Butyrate is a major metabolite in the digestive tract, arising from bacterial fermentation of dietary fibers, mainly “resistant” starch (120, 121), and it is widely available as a dietary supplement. Butyrate modulates gene expression by selectively inhibiting HDAC1 and HDAC3, and, less effectively, other members of class I and class IIa HDACs (117). SCFA HDIs have been suggested to display significant selectivity for different HDACs (122). HDAC activity is mostly associated with multiprotein complexes, the role and composition of which are often cell type-specific. HDAC-associated proteins would specify the selectivity of HDI, which display different affinities for different HDAC/co-factor complexes. HDIs with diverse chemical properties target different HDACs and HDAC/co-factor complexes, thereby regulating gene expression in a locus- and cell type-specific fashion (122). In B cells, HDIs would modulate miRNAs selectively, possibly as a result of HDACs existing in unique contexts of HDAC/co-factor complexes, as occurring in these lymphocytes, particularly when in an activated state (10).

**Figure 2:**
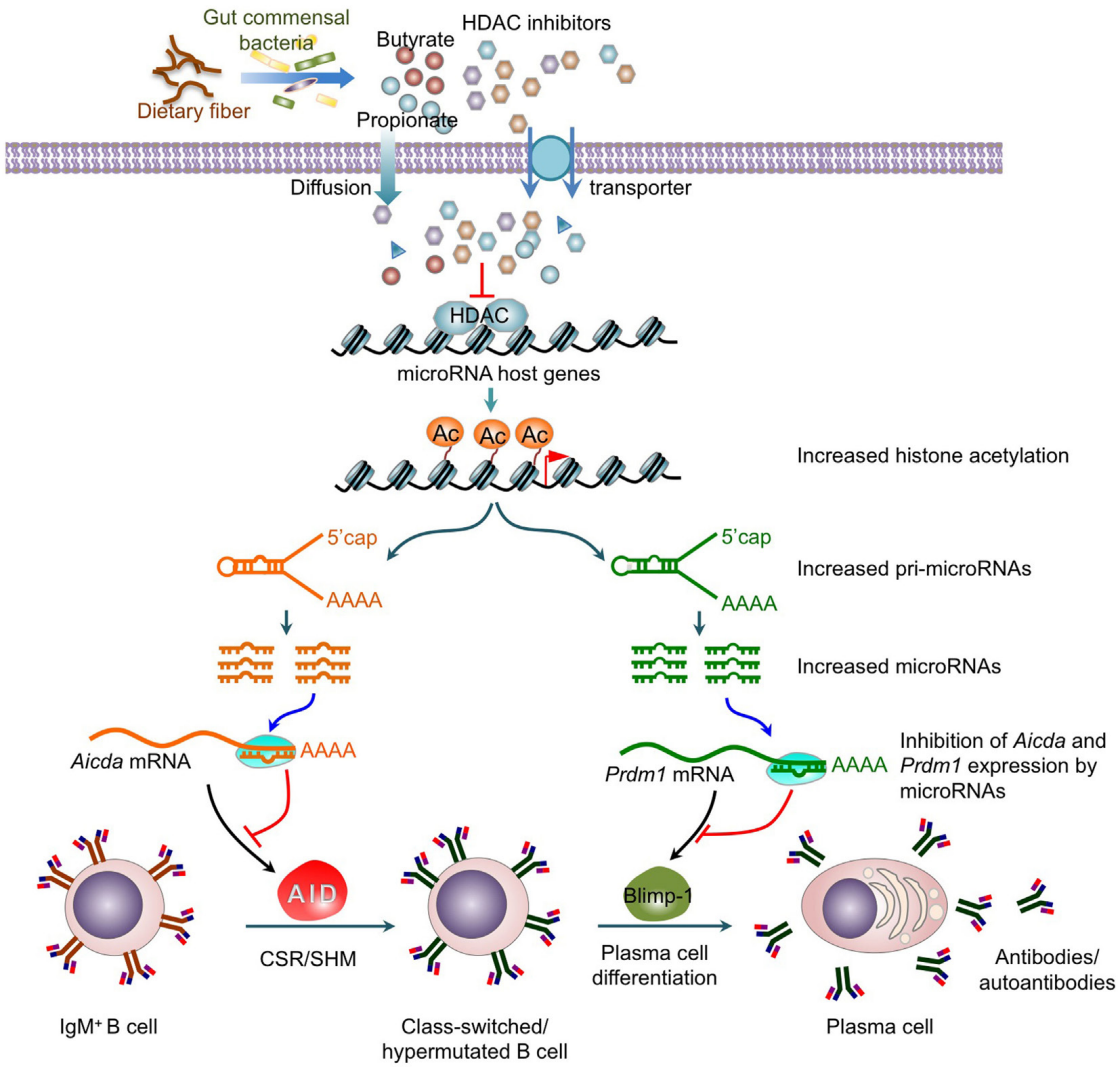
**Histone deacetylase inhibitors upregulate selected B cell miRNAs that silence AID and Blimp-1 expression to epigenetically modulate CSR, SHM, plasma cell differentiation and antibody/autoantibody responses**. HDAC inhibitors, including short-chain fatty acid butyrate and propionate produced by gut commensal bacteria through fermentation of dietary fiber, epigenetically modify CSR and SHM by upregulating miRNAs, which silence *AICDA/Aicda* mRNA and *PRDM1/Prdm1* mRNA. The upregulation of miRNA expression results from an increase in the histone acetylation of the host genes of these miRNAs. This leads to down-regulation of AID and Blimp-1 expression, and the dampening of CSR, SHM, and plasma cell differentiation.

Although HDIs may also indirectly modulate antibody responses or diminish autoimmunity by affecting elements other than B cells, such as innate immune cells (123) and T cells (Treg, T_H_1, and T_H_17 cells), or inhibit proinflammatory cytokines (115, 124–126), HDIs would directly regulate B cell genes that are central to the peripheral differentiation of these lymphocytes and the maturation of antibody and autoantibody responses (Figure 2) (10). Silencing of *AICDA/Aicda* and *PRDM1/Prdm1* (and *XBP1/Xbp1*) by HDIs has been found to be intrinsic to B cells and independent of other cellular elements, as shown by our *in vitro* experiments using purified human and mouse B cells, as well as our *in vivo* studies of the T-dependent response to NP-CGG and the T-independent response to NP-LPS. In both *in vivo* and *in vitro*, HDI-mediated down-regulation of *AICDA/Aicda* and *PRDM1/Prdm1* expression was associated with a concomitant increase of the respective B cell targeting miRNAs (miR-155, miR-181b, and miR-361 for *AICDA/Aicda*; miR-23b, miR-30a, and miR-125b for *PRDM1/Prdm1*), in a tight dose-dependent fashion (10). HDI-induced down-regulation of *XBP1/Xbp1* could be secondary to decreased Blimp-1 expression.

The selectivity of HDI-mediated silencing of *AICDA/Aicda* and *PRDMI/Prdm1* in B cells that were induced to undergo CSR and plasma cell differentiation was demonstrated by genome-wide mRNA-Seq and further emphasized by the unchanged expression of *Ung*, *Irf4*, *HoxC4*, *Rev1*, and *Bcl6*, as well as the unchanged expression of miR-19a/b, miR-20a, and miR-25, which are not known to regulate *AICDA/Aicda* or *PRDMI/Prdm1* (10, 99). This, however, cannot rule out the possibility that HDI regulated other B cell factors (e.g., NF-κB, Id2/3, or Pax5), which contributed to the reduction of AID or Blimp-1. Nevertheless, relief of the HDI-mediated repression of luciferase activity under the control of *Aicda* and *Prdm1* mRNA 3′ UTRs bearing mutated miR-155, miR-181b, miR-23b, miR-30a, and miR-125b target sites demonstrated that miRNAs are indeed direct effectors of the HDI-mediated repression of such selected genes in B cells (10).

### Potential Role for Gut Microbiota-Derived Short-Chain Fatty Acid HDAC Inhibitors in the Modulation of Antibody Response

At ant time, the human body carries 10^13^–10^14^ microorganisms, a number 10-fold more than the total number of human cells in the body. Human gastrointestinal tract microbiota composed of up to 1,000–1,150 bacterial species, which play an important role in nutritional, metabolic and physiological processes that are crucial for the maintenance of human health. Gut commensal bacteria are critical regulators of health and disease by protecting against pathogen while also maintaining immune tolerance to allergens (127–131). Commensal bacteria may modulate host immunity through metabolite-dependent mechanisms (129, 131). SCFAs, such as acetic acid, propionic acid, and butyric acid, which are generated in the colon by commensal bacteria through digestion of dietary fiber, are among the most abundant of these dietary metabolites. They are important for gut motility and colonocyte development. SCFAs function through binding to host cell surface receptors, such as GPR41, GPR43, and GPR109A, and through their HDI activity (132). It has been suggested that SCFAs produced in the gut could distribute systemically and shape the immunological environment in the respiratory system, thereby influencing the severity of allergic inflammation (132).

Mice fed a low-fiber diet displayed decreased serum levels of SCFAs and increased IgE-mediated allergic inflammation in the lung, while a high-fiber diet increased levels of SCFAs and were protected against allergic airway disease (132). Butyrate and propionate, which are potent HDIs, modulate the function of intestinal macrophages and naive T cells to promote epigenetic changes that regulate the expression of genes responsible for differentiation into regulatory T cells and IL-10-producing T cells (121, 132, 133). Our recent findings that butyrate modulates AID expression and CSR to IgG, IgA, and IgE, as well as plasma cell differentiation through its direct HDI activity on B cells (10), indicates that this SCFA can play an important role in modulating antibody responses of gut lymphoid organs. A diverse microbial population, which would produce an appropriate amount of SCFA HDIs, particularly, butyrate, is required to maintain a baseline immune-regulatory state, including IgG, IgA, and IgE levels. Elevated serum IgE and CSR to IgE in B cells at mucosal sites in the absence of microbial colonization in germ-free mice and in mice with low-diversity gut microbiota (134) further emphasize the important role for gut commensal bacteria-produced butyrate in modulating IgE production. Altered composition and decreased bacterial diversity of gut microbiota would lead to changes in absolute and relative levels of SCFA HDIs and, therefore, changes in systemic IgG, IgA and IgE levels and specificities, which contribute to altered immunity and increased susceptibility to immune-mediated diseases.

## B Cell Epigenetic Dysregulation in Autoimmunity and Lymphomagenesis

**Figure 3:**
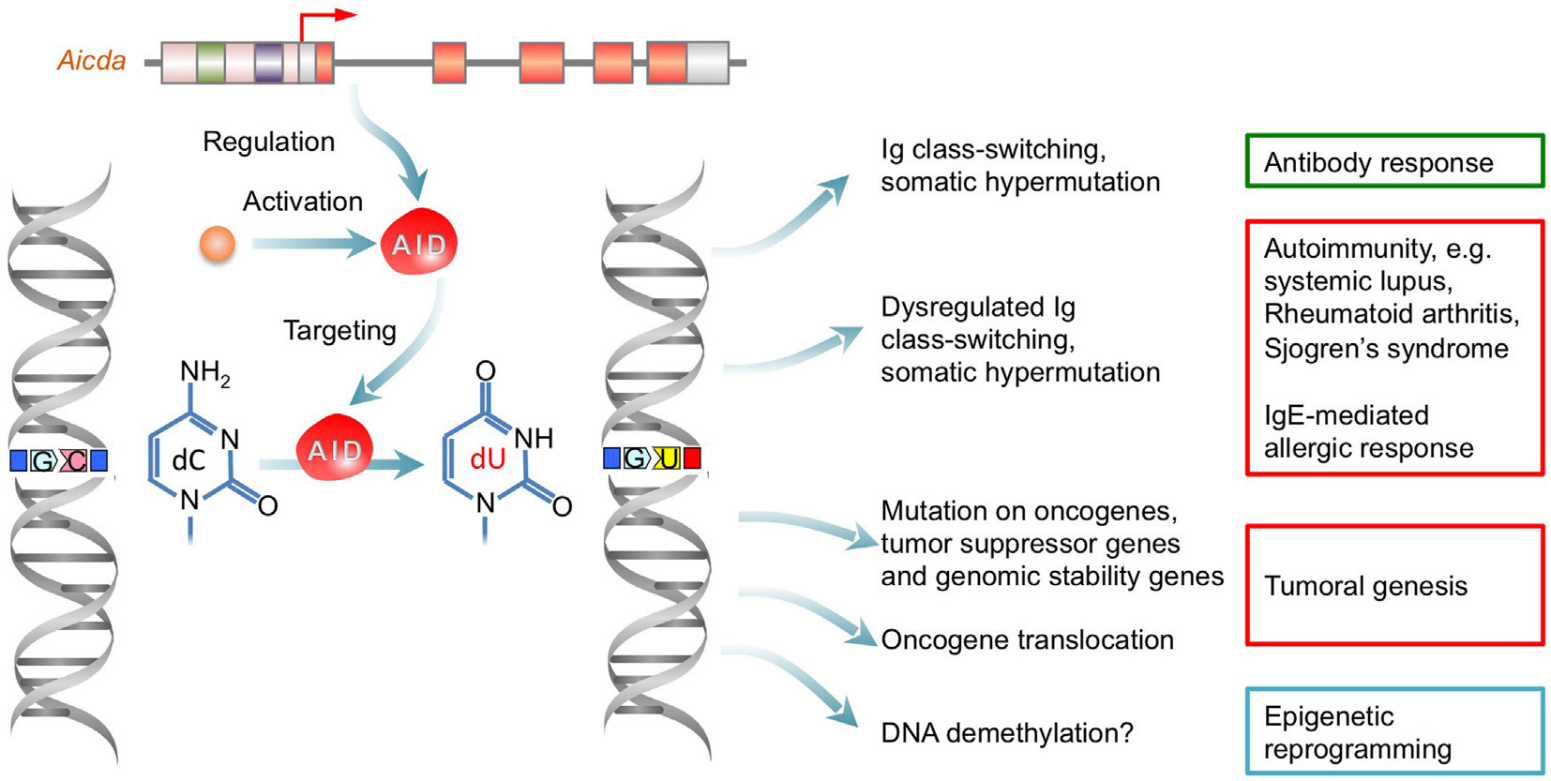
**Dysregulation of AID result in autoantibody-mediated autoimmunity, IgE-mediated allergic response and tumoral genesis**. AID initiates CSR and SHM by deaminating dCs into dUs yielding dU:dG mismatches, which lead to point-mutations in Ig V(D)J regions and DSBs in S regions. Aberrant AID expression result from epigenetic dysregulation can lead to a dysregulated antibody/autoantibody response and AID-mediated DNA mutagenesis, which can cause autoimmunity, allergic response, or tumoral genesis.

Epigenetic factors also play an important role in the pathogenesis of B cell-related immune disorders, such as autoimmunity, allergic states and B cell malignancies, by integrating the effects of genetic makeup and the environment, two major disease-causing factors. Epigenetic dysregulation would compound genetic susceptibility in the generation of autoantibodies and autoimmunity, as suggested by alterations of histone modifications and DNA methylation in patients with lupus, autoimmunity in mice with miRNA dysregulation (9), and low penetrance (25–45%) of lupus in monozygotic twins (135). The development of allergic diseases has been associated with environmental conditions, such as diet, drugs, toxins, sex hormones, and microbiota, all of which can impact the epigenetic profile (136, 137). Aberrant AID expression that can result from epigenetic dysregulation can lead to autoantibody-mediated autoimmunity, IgE-mediated allergic responses and tumorigenesis (Figure 3).

### Epigenetic Changes and Autoimmunity

Systemic autoimmune diseases, such as SLE, rheumatoid arthritis, systemic sclerosis, and dermatomyositis, are associated with overall DNA hypomethylation (1, 138, 139). Autoreactive B cells in lupus patients are characterized by lack of ability to induce DNA methylation that extends their survival (140). In mice, prolonged treatment with DNA methylation inhibitors, or adaptive transfer of B cells treated with DNMT inhibitors, resulted in autoantibody production and lupus-like disease (141, 142). The role of DNA methylation in autoimmune diseases is further emphasized by the discordance of SLE in monozygotic twins. B cells isolated from SLE patients displayed profound defects in DNA methylation, as compared to those from healthy monozygotic siblings (135). The decreased DNA methylation in autoreactive B cells likely resulted from reduced DNMT1 and DNMT3b expression; it could also result from active DNA demethylation mediated by AID-mediated cytosine deamination (143). Indeed, AID is upregulated in B cells of lupus patients or lupus-prone mice (3, 9, 144). The contribution of aberrant histone modifications to lupus development has been strongly suggested by the increased histone acetylation and reduced autoantibody production in lupus-prone mice treated with HDIs (1, 10, 145).

Dysregulation of miRNAs has been associated with autoimminty. miRNAs are aberrantly expressed in different cell types and tissues in patients with autoimmune disease. B cell-specific deletion of Dicer, which is critical for miRNA maturation, resulted in a distorted BCR repertoire with increased of autoreactivity, suggesting a role for miRNAs in preventing the generation of self-reactive antibodies (9, 146, 147). In lupus-prone MRL/*Fas^*lpr/lpr*^* mice, miR-150 is downregulated in spleen B cells, as compared to that in MRL/*Fas*^+/+^ mice (148), possibly as a result of decreased acetylation and transcription of the miR-150 host gene due to defective HDAC activity. Indeed, specific knock-in dominant negative mutant of histone acetyltransferase p300 in B cells resulted in production of class-switched anti-double-stranded DNA autoantibodies and development of lupus-like symptoms (149). Conversely, elevated expression of some miRNAs can also contribute to the development of autoimmunity. In lupus mice, expression miR-21 is upregulated in B cells, silencing of this miRNA ameliorates autoimmune splenomegaly (150). Constitutively expression of miR-17 ~ 92 in transgenic mice results in an increased numbers of germinal center B cells in the spleen and peripheral lymph nodes that may lead to autoimmune response (151).

### Epigenetics Changes and Lymphomagenesis

At all stages of cancer development, inappropriate epigenetic marks interact with genetic alterations to promote neoplastic transformation and tumor cell progression (152, 153). The epigenome of B cell lymphomas is characterized by global changes in DNA methylation and histone modification patterns, which varies with chromosomal regions, local gene density, as well as DNA and histone modification status of neighboring genes (Table 2) (154, 155). Aberrant DNA hypomethylation of promoters can lead to increased transcription of genes with oncogenic potential, and aberrant DNA hypermethylation can lead to decreased transcription of genes with tumor suppressor function. For example, mantle B cell lymphomas display DNA hypomethylation in promoters of genes that are involved in pathways controlling cell cycle or apoptosis, such as *Cdk5*, and aberrant hypermethylation in the promoter of tumor suppressor genes, such as *Cdkn2b* (156). Hypermethylation is likely associated with increased expression of DNMTs, such as DNMT3b, which is upregulated in diffuse lager B cell lymphomas (157). Accordingly, *Dnmt3b* transgenic mice develop mediastinal B cell lymphomas, which display significantly altered methylation patterns (157). Aberrant hypomethylation could involve AID, which can mediate active DNA demethylation (158) and is upregulated in human B cell non-Hodgkin lymphomas (NHL) (159).

**Table 2:** **B cell epigenetic dysregulation and lymphomagenesis**.

Neoplasm	Epigenetic change	Potential impacts of epigenetic changes	Reference
DLBCL, FL	Loss-of-function mutation of HAT genes *CREBBP* and *EP300*	Reduce P53 and BCL6 acetylation	(177, 178)
HD, NHL	Deregulation of the H3K27 methyltransferase EZH2	Malignant GC B cell transformation	(179)
Multiple myeloma	Overexpression of H3K36 methyltransferase *MMSET/NSD2*	Alter H3K36 and H3K27 methylation, upregulate *c-MYC* expression by reducing *c-MYC* targeting miR-126	(180)
NHL, DLBCL, FL, MLL	Dysregulation of the H3K4 methyltransferase MLL2/MLL3/KMT2D	Modulate cell-type- and stage-specific transcriptional programs by regulating chromatin accessibility at enhancer regulatory sequences	(181)
NHL, DLBCL	Aberrant DNA methylation	Promote aberrant gene expression	(155, 182, 183)
B cell CLL	miR-15a/16-1 depletion	Aberrant expression of cell cycle regulators Ccnd2, Ccnd3, Cdk4, Cdk6 and Chk1, and anti-apoptotic protein BCL2	(171, 184)
NHL, HD, BL	miR-155 deregulation	Enhance/sustain AID mutagenic activity	(37, 185)
DLBCL, BL	Overexpression of the miR-17-92 cluster	Downregulate inhibitors of the PI3K (*Pten*), NF-κB (*A20, Cyld*) and the intrinsic apoptotic (*Bim*) pathways; alter MYC-centered regulatory network	(151, 186, 187)

Active DNA demethylation can also be initiated by the 10–11 translocation (Tet) family of proteins Tet1, Tet2, and Tet3, which catalyze the oxidation of 5mC to 5-hydroxymethylcytosine (5hmC), a critical step for ultimate removal of a methyl mark (160, 161). Reduced expression of Tet proteins that lead to decreased 5hmC has been shown to associate with tumor development, suggests an important role of Tet proteins and 5hmC in normal cellular function (162). Tet1 is required for maintaining the normal abundance and distribution of 5hmC, which prevented hypermethylation of DNA in B cells (163). It is important for regulation of the B cell lineage and of genes encoding molecules involved in chromosome maintenance and DNA repair. Tet1 may function as a tumor suppressor of B cell malignancy. Deletion of *Tet1* in mice promotes the development of B cell lymphoma (163).

Altered expression or mutation of the histone-modifying enzymes promotes aberrant gene expression, which is responsible for many tumor changes (164). Aberrant germinal center development is common in many B cell malignancies. EZH2, a histone methyltransferase component of polycomb repression complex (PRC)2 that catalyzes H3K27me3 and promotes tumor growth, is highly expressed in germinal center B cells and is often constitutively activated in germinal center-derive NHLs. EZH2 prevents apoptosis caused by DNA damage, including that generated by AID, facilitated cell cycle progression, and silenced Blimp-1, which can function as tumor suppressor (106). Inhibition of EZH2 in NHL cells induces Blimp-1, which impairs tumor growth. Overexpression of EZH2 is associated with B lymphomagenesis (165). Somatic mutations at Y641 and A677 residues within the catalytic domain of EZH2 have been found in diffuse large B cell lymphoma and follicular lymphoma (166). These mutations promote EZH2 activity and increase H3K27me3 levels in those cells (165). Furthermore, other histone-modifying enzymes, such as histone acetyltransferases CBP and p300, histone methyltransferases MLL2, and histone demethylases UTX and JMJD2C, are frequently mutated in B cell lymphomas (157). Mutations in these enzymes are likely mediated by AID, which is highly expressed in those neoplastic B cells, and result in aberrant patterns of histone modifications and disruption of chromatin structure, ultimately leading to dysregulated gene transcription programs.

Dysregulated miRNA expression also contributes to B lymphomagenesis (28, 167). The miR-17 ~ 92 cluster, which consists of six miRNAs (miR-17, miR-18a, miR-19a, miR-20a, miR-19b-1, and miR-92-1) that target the tumor repressor genes *Bim* and *Pten*, is often increased in human lymphomas (151, 168). Accordingly, ectopic expression of the miR-17 ~ 92 cluster in lymphocytes leads to development of lymphoproliferative disease (151, 168). Conversely, expression of miR-15 and miR-16-1, which target anti-apoptotic oncogene *Bcl-2*, is downregulated in chronic lymphocytic leukemia B cells (169, 170). Knockout miR-15a and miR-16-1 in B cells results in clonal lymphoproliferative disorders (171). B cell lymphomas often express significant amounts of mRNA isoforms with shorter 3′ UTRs, which lack miRNA-binding sites, thereby escaping miRNA-directed silencing (172). For example, a short *Cyclin D1* mRNA isoform that lacks part of the 3′ UTR is expressed in a subset of mantle cell lymphomas (172). These cells have increased expression of Cyclin D1, an important regulator of cell cycle progression, and B cell proliferation. Thus, by allowing selected oncogenes to escape regulation by their modulatory miRNAs, the shortening of 3′ UTRs may provide an important mechanism in B cell neoplastic transformation.

## Concluding Remarks

Epigenetic changes are critical in shaping B cell differentiation functions, such as CSR, SHM, generation of plasma cells as well as memory B cells, for the production of class-switched and high affinity antibodies. In addition, a growing body of evidence implicates the involvement of epigenetic mechanisms in immune programing and development of allergic and autoimmune diseases. Nevertheless, important questions on the nature and role of such epigenetic changes remain to be answered. These include the mechanisms by which histone posttranslational modifications and non-coding RNAs target the CSR and SHM machineries, particularly the selective recruitment of AID, to the *Ig* locus and how the dynamics of these epigenetic modifications orchestrate AID-mediated DNA lesion and DNA repair processes. High-affinity antibodies are generated in germinal centers through SHM and selection of higher affinity B cell submutants for survival and expansion. These B cells will then undergo differentiation to plasma cells or memory B cells. Much needs be understood on the role of epigenetic modifications in the selection of germinal center B cells and in what determines whether a germinal B cell becomes a memory B cell or a plasma cell. These processes would involve unique epigenetic and transcriptional changes. Furthermore, altered DNA methylation, histone methylation and acetylation, and miRNA expression, resulting in immune imbalance, have been shown to be associated with the onset and progression of allergic and autoimmune diseases, as well as B cell lymphomagenesis. Thus, knowledge of the epigenetic profiles associated with B cell development and peripheral differentiation, and molecular mechanisms that cause and result from disease-associated epigenetic patterns in B cells is required to understand the pathophysiology of allergic and autoimmune diseases, as well as B cell malignancies.

## Conflict of Interest Statement

The authors declare that the research was conducted in the absence of any commercial or financial relationships that could be construed as a potential conflict of interest.
